# Effects of Environmental Features in Small Public Urban Green Spaces on Older Adults’ Mental Restoration: Evidence from Tokyo

**DOI:** 10.3390/ijerph19095477

**Published:** 2022-04-30

**Authors:** Shan Lu, Wonseok Oh, Ryozo Ooka, Lijun Wang

**Affiliations:** 1 Department of Architecture, School of Architecture, Tianjin University, Tianjin 300072, China; lushan0710@tju.edu.cn; 2 Institute of Industrial Science, The University of Tokyo, Tokyo 153-8505, Japan; oh-ws@iis.u-tokyo.ac.jp (W.O.); ooka@iis.u-tokyo.ac.jp (R.O.)

**Keywords:** small public urban green spaces (SPUGS), mental restoration, environmental features, thermal comfort, older adults

## Abstract

Exposure to small public urban green spaces (SPUGS) has been demonstrated to have mental benefits for older adults. However, studies on identifying the objective environmental features of SPUGS and their effects on mental restoration for older adults remain limited. This study employed a multilevel regression model to investigate the restorative and vitalizing effects of the environmental features of 11 SPUGS in Tokyo. Onsite measurements were conducted in Kita-Ku, and 202 older adults were surveyed. The results showed that: (1) The fitting curve of the green view index and Restoration Outcome Scale (ROS) score showed an inverted U shape—both green view index and boundary enclosure had a strong impact on the mental restoration of older adults; (2) The colorfulness index showed the strongest relationship with the vitalizing effect. (3) The sky view factor and number of seats only influenced the ROS score, while the results of revitalization suggest that large areas of water should be avoided. (4) Physiological Equivalent Temperature (PET) was also confirmed to have negative effects on the mental restoration of older adults in autumn. These empirical findings can be used as a resource to promote the mental health of older adults in the design of SPUGS in high-density Asian countries.

## 1. Introduction

Japan faces a dual challenge with an advanced aging society and population reduction. The proportion of the population over 65 years old is predicted to reach 35.3% in 2040, while the total birth number continues to decline and has reached 740,000, which is about 90% of that in 2019 [1]. Population reduction has resulted in the destruction of the traditional family, which can cause the experiences of loneliness among the urban residents [2]. Combining this with smaller social networks and more functional limitations [3], the elderly population faces serious mental health challenges today. The number of Japanese older patients (≥65 years old) with mood or anxiety disorders reached 312,300, accounting for 69.89% of the total hospitalized mental patients in 2017 [4]. Further, under the global spread of COVID-19, social distancing measures have raised concerns about older adults’ mental health [5]. In a study conducted in seven prefectures in Japan, 25.2% of the older adult respondents were suffering from mild to moderate psychological distress during the pandemic period [6].

Higher exposure to urban green spaces (UGS) is associated with longevity [7], the enhancement of physical activity [8,9,10], social connection [11], and the relief of stress-related illnesses such as depression [12,13] in older adults, especially if they are situated close to their homes [14]. Most existing studies have focused on larger green spaces (e.g., city parks, riverside green spaces, and forests); however, smaller UGS in high-density areas have recently received more attention [15,16,17]. Karin Peschardt et al. [18] defined small public urban green spaces (SPUGS) as public spaces not exceeding 5000 m^2^ in size with some vegetation and their own entrance. Studies have reported on the vital role of SPUGS in promoting residents’ mental and physical health [19]. Based on the Attention Restoration Theory (ART) [20] and the Stress Reduction Theory (SRT) [21], SPUGS is primarily used for “socializing”—the act of meeting for social purposes—and “rest and restitution”—the process of restoration from mental fatigue [18,22]. However, research on SPUGS is still too limited to be translated into concrete design strategies appropriate for older adults’ unique physiological characteristics. Moreover, most studies have been conducted in Western contexts [23].

Tokyo’s efficient UGS system makes it livable and accessible for its high-density aged population; therefore, the way in which the SPUGS environmental features influence older adults differs from its effects in a Western context. What can and should be done by landscape designers so that SPUGS meet the requirements of and improve older adults’ mental health? Which of the environmental features of SPUGS have an impact on the older adults’ mental restoration outcomes? What is the impact of each related variable? These questions require immediate attention, particularly in high-density Asian countries.

### 1.1. Environmental Features in SPUGS

Studies have attempted to identify the specific objective variables of UGS that influence the psychological improvement of older adults. The accessibility of SPUGS has been a critical issue [24]. The distance from one’s home to the green space in the neighborhood is related to the psychological stress recovery benefits for the older adults, especially those from a low socioeconomics class [25,26]. Euclidean distance and self-reported walk time have been commonly applied in studies [27,28]. Natural features attract people to outdoor activities and thus, promote their psychological well-being. Nordh and Ostby [22] found that natural components such as “a lot of grass/plants” and “water features” in small urban parks can promote opportunities for restorative experiences. Research on the plantings in UGS has been divided into three layers: “ground cover,” “eye-level green,” and “tree canopies” [19,29].

Furthermore, the restorative benefits of “green ground cover” and “eye-level greenness” in pocket parks have been confirmed to be the opposite of those of large UGS for mental promotion [19]. The “green view index”, an indicator of eye-level visibility of green vegetation [30,31], could represent the actual feelings of green space users in environmental research and public health studies [32,33]. The spatial form design of UGS may influence how participants perceive and interact with the space and, thus, may affect their mental health [34,35].

Chen et al. [36] observed that spatial transparency and spatial enclosure were significantly correlated with older people’s moods in poor-quality residential areas. The width of the adjacent street and the average height of surrounding buildings in small-scale street corner spaces also impact the frequency of seniors’ communication behavior, thus affecting their mental well-being [37]. Meanwhile, Chen and Zhang [38] demonstrated that a space’s layout, aesthetic, and recreational services influenced study participants’ appraisals of their pleasure, arousal, and control. 

The Environmental Assessment of Public Recreation Spaces tool (EAPRS) [39] and the Neighborhood Green Space Tool (NGST) [17] have been used in various studies. Both of them emphasized the importance of the quality of UGS facilities (e.g., paved and unpaved trails, tables, benches, cafés, playgrounds). In relation to microclimate features, thermal comfort has been observed to be significantly correlated with psychological restoration outcomes using physiological equivalent temperature (PET) and predicted mean vote (PMV) [40,41] in roadside and forest scenes. However, with the degeneration of thermal sensation in older adults [42], the mental restoration of objective environmental features can vary with age, resulting in the restoration benefits having different effects. Moreover, most of the research perspectives have been relatively singular. How to systematically present the effect of the different dimension features in SPUGS on the mental restoration benefits of older adults remains a problem that needs to be solved.

### 1.2. Mental Restoration of Older Adults

Experimental evidence shows that visiting or seeing natural elements alleviates attentional fatigue and emotional stress [43,44,45,46]. The Restoration Outcome Scale (ROS) can be widely used to investigate restorative emotional and cognitive outcomes in a given environment [40,47,48]. ROS is confirmed to be reliable in evaluating the participant’s relaxation, attention, and calmness when exposed to nature and green spaces [46]. With regard to mental responses in SPUGS, the self-reported vitality restoration score was considered to be the result of contact with nature in parallel with the ROS. Subjective vitality is related to mental status [49,50,51].

In contrast to low energy states (e.g., relaxation), it reflects high energy states (aliveness, energy available for self) and is positively related to mental [50] and physical health [52]. Subjective vitality enables older adults to maintain a physically and socially active life, which can delay physical and cognitive decline associated with aging [53]. Several studies conducted in different countries and locations have reported that the presence of outdoors or natural environments provides better vitality experiences [48,54]. Based on these studies, it seems that SPUGS have the potential to be areas for subjective vitality recovery. In this study, we are interested in investigating which of the environmental features of SPUGS affect the elderly’s subjective vitality restoration.

### 1.3. Research Framework

**Figure 1:**
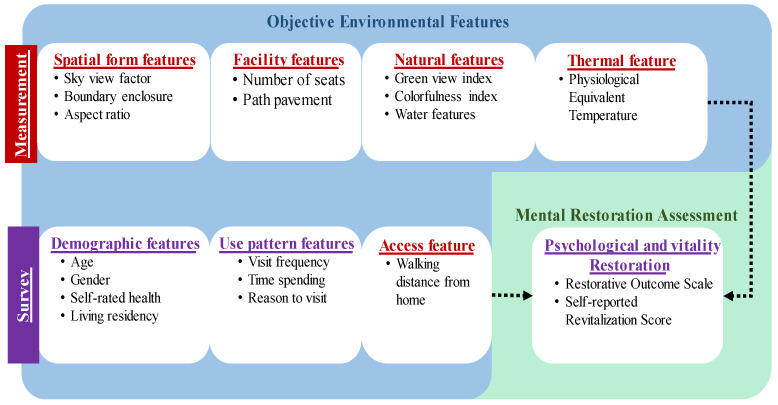
Research framework.

To help fill the gaps in the literature mentioned above, this study integrates access, spatial form, facility, nature, and the thermal features of SPUGS. We aim to identify the effect of the key environmental features of SPUGS on the mental restoration of older adults by controlling for individual indicators. The conceptual framework is shown in Figure 1. The study is expected to advance our current understanding of how the key SPUGS features of access, spatial form, nature, facility, and thermal conditions influence its restorative health benefits for seniors.

## 2. Materials and Methods

### 2.1. Overall Research Procedure

**Figure 2:**
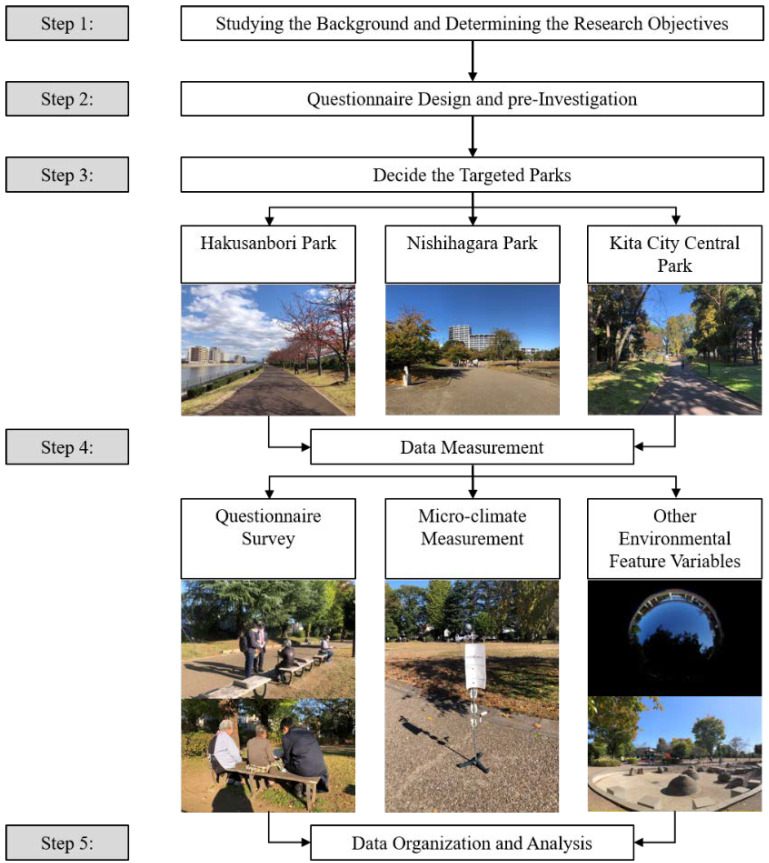
Research procedure.

The research procedure comprised five steps corresponding to specific measurement methods and tasks (Figure 2). The target area for this study was determined through a background survey of aging population information in Tokyo (Step 1). Thereafter, the questionnaire survey was designed and examined by experienced professors and researchers in related fields (Step 2). A pre-investigation was conducted in all parks located in Kita-ku, Tokyo, in order to target the research to specific parks based on the following criteria: (1) parks with different percentages of an overhead tree canopy, (2) parks with various different functions, and (3) parks located in zones with different aging rates (percentage of people over 65 within the total population of Kita-Ku) (Step 3). This formal investigation was conducted from 6–15 November 2020 (10 a.m.–3 p.m.). The questionnaire survey, microclimate measurement, and the recording of other environmental features were conducted simultaneously (Step 4).

To record the climatic conditions experienced by the respondents when they completed the questionnaire, the investigators were equipped with data loggers to measure air temperature and relative humidity upon arrival at the parks. To ensure that the respondents’ feelings were accurately recorded, the investigators selected senior participants who had been in one space for no less than five minutes and noted the time and location (whether in a sunny or shaded area) when they finished. Finally, all the data were organized and analyzed (Step 5).

### 2.2. Selection of Targeted Parks and SPUGS Typologies

**Figure 3:**
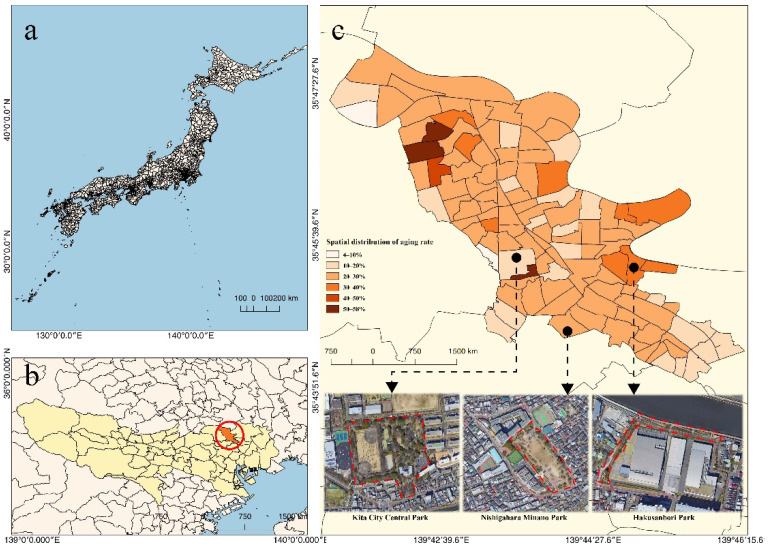
Spatial distribution of the aging rate in Kita-ku, Tokyo. They are (**a**) The full view of Japan; (**b**) The location of Kita-ku in the Tokyo Metropolitan; (**c**) The spatial distribution of the older adults in Kita-ku and the location of three targeted parks.

Tokyo locates in the humid subtropical climate zone, and the weather in November is usually mild with an average temperature of 13.3 °C. It is well-known that November is the best season to walk outside and enjoy the tree leaves in their glory of russets, reds, and browns in Tokyo. The average aging rate of Kita-Ku is 25.5%, which is significantly higher than the average rate across Tokyo (22.1%). Figure 3 illustrates the spatial distribution of the aging rate in Kita-Ku. Based on a preliminary investigation, three parks were chosen: Hakusanbori Park, Nishigahara Minnano Park, and Kita City Central Park. The basic information about each of the selected parks is shown in Table 1. The spatial aging rates of the three selected parks are 36.27%, 25.10%, and 21.00%. Hakusanbori Park is a neighborhood park with an area of 2500 m^2^. Nishigahara Minnano Park is a city block park with a large proportion of lawn-grass coverage acting as the playground for residents’ leisure time. Kita City Park is a comprehensive park for education, tennis, baseball, and walking.

Due to older adults’ limited mobility, they may not walk the entire park; therefore, each park was divided into two or three SPUGS that older adults often use or stay in to better understand the parks’ environmental features and prevent regional and statistical bias. The characteristics of 11 SPUGS are shown in Appendix A.

### 2.3. Environmental Factors and Variable Measurements

#### 2.3.1. Microclimate Measurement

**Figure 4:**
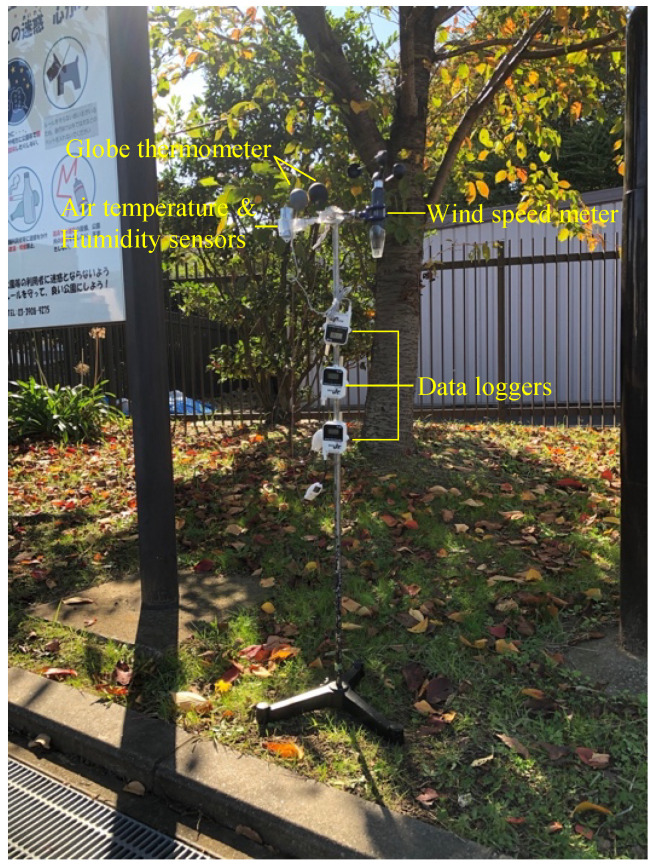
The fixed weather station.

During the onsite measurement, air temperature (°C), relative humidity (%), global temperature (°C), and wind velocity (m/s) were recorded. The results and the data collection equipment used are listed in Table 2. Due to the daily behaviors of older adults, the measurements were carried out between 9 am and 3 pm. Measurement points were selected in sunny and shaded areas within the target parks in order to reflect typical climatic conditions. Microclimate measurements were taken over two days in each park. The sensors were all placed in one fixed weather station, and all instruments were placed at a height of 1.1 m above ground (Figure 4). The globe temperature was measured using a globe thermometer with a 40 mm gray table-tennis ball and a *T*-type thermocouple. Appendix A lists the weather conditions during the study days.

PET is defined as the air temperature at which, in a typical indoor setting (without wind and solar radiation), the heat budget of the human body is balanced with the same core and skin temperature as under the complex outdoor conditions to be assessed [55]. It was selected as a variable to represent human thermal comfort in this study. The mean radiant temperature ($T_{\mathrm{mrt}}$) was calculated according to Equation (1), as developed by ASHRAE (2017a) and refined by Thorsson et al. [56].

(1) $$T_{\mathrm{mrt}}=\left[\frac{{\left(T_{g}+273\right)}^{4}+\left(1.10*10^{8}v^{0.6}\right)\left(T_{g}-T_{a}\right)}{\varepsilon D^{0.4}}\right]-273$$

where $T_{\mathrm{mrt}}$ is mean radiant temperature (°C), $T_{g}$ is globe temperature (°C), $T_{a}$ is air temperature (°C), v is air velocity (m/s), D is global diameter (m), and ε is the emissivity of the sphere. The metabolic rate was set to 1.2. Based on the clothing insulation information collected in the questionnaire survey, a series of analyses were conducted with MATLAB software.

#### 2.3.2. Spatial Form, Nature, and Facility Feature Variables

**Figure 5:**
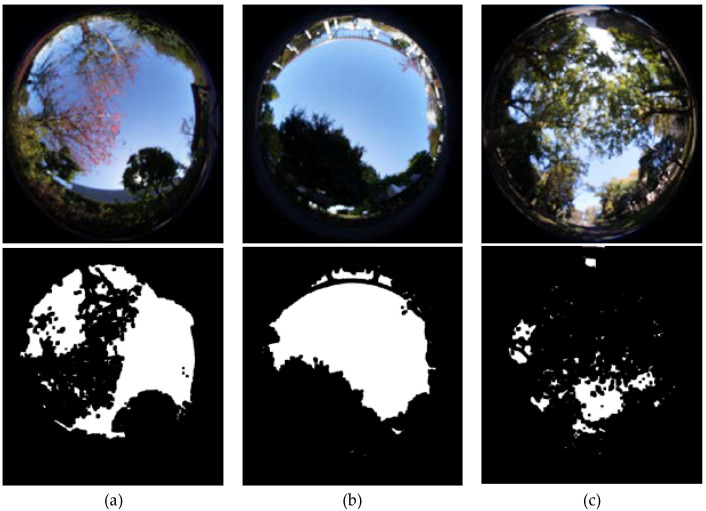
Examples of skyview factor calculation by using image semantic automatic recognition technology and statistic of methods for the three parks. They are (**a**) Hakusanbori Park; (**b**) Nishigahara Minnano Park; (**c**) Kita City Park.

Each research unit was divided into the bottom, horizontal, and top interfaces. Three spatial form variables were selected: sky view factor, aspect ratio, and boundary enclosure. The sky view factor was measured using a fish-eye lens (4 mm, F2.8) camera at a height of 0.7 m above ground. The proportion of sky-colored pixels was calculated in Python code using image sematic automatic recognition technology and statistical methods (Figure 5). The sky view factor was measured once in the morning during the measurement period at the same point where the older respondents were present. The trails’ sky view factor was the mean sky-colored pixels of each picture taken every 5 m. Digital models of all sites were built in the ArcGIS software, based on data collected from the aerial view images on Google Maps. Onsite measurements were also taken to compensate for aspect ratio, and boundary enclosure. The aspect ratio was calculated as the ratio of the average height and width of the area. The boundary enclosure was graded by “1 = unilateral enclosure”, “2 = parallel enclosure”, “3 = u-shaped enclosure”, “4 = almost complete enclosure”.

We chose the parks’ green view index, colorfulness index, and water feature as the natural feature variables according to the description of the NGST [17]. The green view index was calculated using the proportion of green pixels in photos taken at the human eyeline. The purpose of the colorfulness index is to calculate the natural color richness of the space in the field of vision using python code. The correspondence between the colorfulness metric and the colorfulness attributes refers to the research of Hasler and Suesstrunk [57]. The research space with or without water features was observed by our investigators with a “yes” or “no” value.

Considering Japan’s neighborhood park conditions, we chose to examine the trail pavement and quantity of benches as facility variables. The trail pavement condition was recorded with a “paved” or “unpaved” value. The number of benches in each research unit was also recorded.

### 2.4. Questionnaire Survey

The questionnaire data was collected with the help of six students. The questionnaire survey comprised three parts. The first asked about the participants’ demographic information (age, sex, self-rated health, length of residency), their use patterns (length of stay in the park, use frequency, the reason for visit), the level of clothing worn (the checklist modified from ASHRAE [58]), and the access feature of the SPUGS (self-reported walking distance from home).

The second part of the questionnaire assessed the psychological restoration and subjective vitality benefits of the spaces via the ROS, which is a reliable and valid scale to evaluate the restorative emotional and cognitive outcomes of nature and green spaces [40,48], as well as the self-reported vitality recovery score. The ROS includes six items, each of which is evaluated using a seven-point Likert scale (from 1: “strongly disagree” to 7: “strongly agree”). The Japanese version of the ROS questionnaire used by Fujisawa and Takayama [59] was adopted in the present study. Participants’ feelings regarding the restoration of their vitality were also evaluated; taking into account the time taken to complete the questionnaire and the subjects’ patience, we used the sentence, “I feel alive and vital after being in this place,” which was rated on a 7-point Likert scale of agreement (from 1: “strongly disagree” to 7: “strongly agree”).

### 2.5. Statistical Analysis

Due to the hierarchical nature of the data (the questionnaire survey was conducted at the individual level, while the parks’ environmental feature variables were investigated at the neighborhood level), a multilevel regression model was used to investigate the effects of key environmental feature variables that impacted the older adults’ restorative and vitalizing effects in the SPUGS. Null models were conducted by separately taking the ROS and subjective vitality scores as the dependent variables. This step was mainly used to test whether the SPUGS environmental features affected the psychological and subjective vitality benefits to older adults. It was used to determine the necessity of a multilevel regression model, see Equations (2) and (3).

First, the individual features were added to explore their effects at the individual level. The access, spatial form, nature, facility, and access features, as well as the thermal condition indices of each research unit, were then fitted into the full model to examine their effects. A smaller AIC implies a better model. The different models were then fitted to the hierarchical data using STATA 14.0 (StataCorp LLC, Texas, USA).

(2) $$L_{individual}:\;y_{ij}=\beta_{0j}+\epsilon_{ij}$$

(3) $$L_{neighborhood}:\;\beta_{0j}=\gamma_{00}+\delta_{0j}$$

where $y_{ij}$ represents the ROS and subjective vitality score separately. $\beta_{0j}$ represents the intercept of the SPUGS model. $\epsilon_{ij}$ is a random variable at the individual level. $\gamma_{00}$ represents the overall mean value of the older adults’ ROS and subjective vitality score. $\delta_{0j}$ represents a random variable at the community level. If $\delta_{0j}$ is statistically significant, a hierarchical model is required.

The prediction of SPUGS’s mental restoration benefits from regression of ROS and subjective vitality score on significant environmental features’ continuous variables were calculated in STATA 14.0 to investigate the influence trend and find the optimal threshold of the related variables.

## 3. Results

### 3.1. The SPUGS’s Environment Features and Respondents’ Characteristics

A total of 202 older adults aged over 60 years participated in this study: 66 in Hakusanbori Park (Park A), 70 in Nishigahara Minnano Park (Park B), and 66 in Kita City Central Park (Park C). The respondents’ individual features, ROS, and subjective vitality scores are presented in Table 3. Male participants outnumbered female participants by 25.74%. Most of the participants reported that they were healthy. Overall, 83.66% of participants declared that they had lived in the neighborhood for more than three years, and 59.90% of them visited the research park once or twice a week. More than half of the senior respondents said they stayed in the park for more than 30 min each visit. Of the participants who visited the park, 42.08% reported that they came for exercise, 27.23% came for relaxation, and 14.85% came with their family or friends for companionship. The average ROS score of the sample was 33.91 ± 4.00 (average ± SD), while the subjective vitality score was 5.13 ± 0.95 (average ± SD). We delineated two groups based on the respondents’ sex. The demographic and usage pattern features were similar to the characteristics of the total sample. Table 3 indicates that female respondents intended to visit SPUGS with family members or friends. The average PET of all respondents is 15.24 °C, while that of females is 0.78 °C higher than that of males. The ROS scores of men were higher than women; the opposite was true for the vitalizing effects.

Table 4 shows the calculations for each of the environmental feature variables of the SPUGS. The sky view factor of c4 (a lawn space) is the highest (1.000), while c6 scored the lowest (0.047). The aspect ratio of c5 ranks the highest among the 10 other spaces at 6.670 since it is a fitness trail in Kita City Park with aligned trees, grass, and shrubs. B3, c1, c2, and c3 received the same boundary enclosure, which is surrounded by aligned buildings, trees, grass, shrubs, and benches. In the natural features category, a2 had the highest score on the colorfulness index among all the spaces, with its excellent natural planting color. In terms of facility features, a2 and c5 had an adequate number of seats and paved roads.

### 3.2. Effects of Individual and Environmental Features on ROS

The results of the effects of individual and environmental features on ROS are presented in Table 5. The null model showed that the environmental variables explained a significant portion of variance (18.3%; ICC = 0.183) in the ROS of the senior respondents. Model 2 indicates the effects of the respondents’ park use patterns and individual features on ROS scores. Firstly, participants who were aged over 80 generally reported higher levels of psychological restoration (Coef. = 1.197; p < 0.05) than those aged 60–69. Secondly, participants who had been in the park space for 10–30 min (Coef. = 2.716; p < 0.01) and over 30 min (Coef. = 3.313; p < 0.001) gained more psychological restoration benefits. Besides, companionship is the most important indicator of older people’s mental restoration (Coef. = 3.405; p < 0.001).

**Figure 6:**
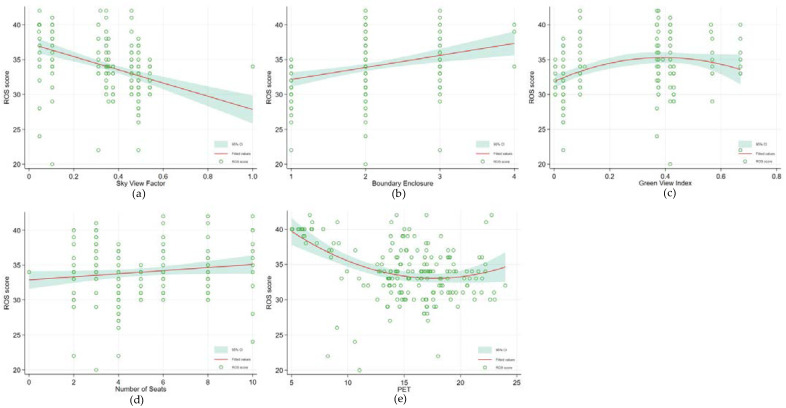
Independent curvilinear associations of environmental feature variables with ROS score. They are (**a**) The independent curvilinear association of sky view factor with ROS; (**b**) The independent curvilinear association of boundary enclosure with ROS; (**c**) The independent curvilinear association of green view index with ROS; (**d**) The independent curvilinear association of seats number with ROS; (**e**) The independent curvilinear association of PET with ROS. [Red lines represent point estimates of modeled score of ROS, while the green background areas represent their 95% confidence intervals].

Access, spatial form, nature, facility, and the thermal condition features of every research unit were added to Model 3. The AIC in Model 3 was smaller than in Models 1 and 2, which indicated that the whole sample model had been greatly improved. The results showed that time spent, 10–30 min (Coef. = 2.971; p < 0.01) and more than 30 min (Coef. = 3.196 p < 0.001), and companionship (Coef. = 2.295; p < 0.001) still significantly impacted the ROS. As for the environmental features, the access feature showed no relation with the ROS score. The sky view factor (Coef. = −7.906; p < 0.05), boundary enclosure (Coef. = 1.901; p < 0.01), green view index (Coef. = −4.800; p < 0.05), and number of seats (Coef. = 0.502; p < 0.001) showed a strong association with psychological recovery benefits (see Table 5 and Figure 6). It can be seen that there is a linear relationship between sky view factor, boundary enclosure, the number of seats, and the ROS score of the older adults (see Figure 6a,b,d). However, the fitting curve results of the green vision rate showed an inverted U shape. After the green view index reached 0.4, the ROS score started to show a downward trend. Therefore, it could be speculated that, a space with a high sky view factor, enclosure of the interface boundary, and enough seats, could provide a sense of security, greenery, and a convenient place to rest, which could benefit the psychological health of older adults.

**Figure 7:**
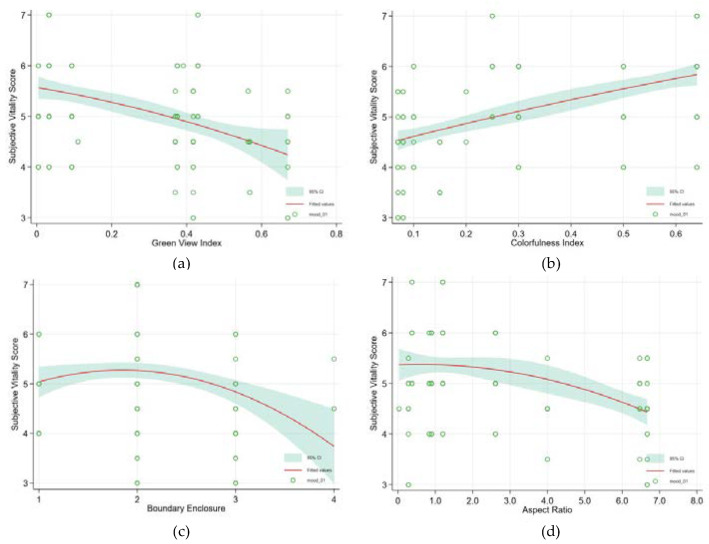
Independent curvilinear associations of environmental feature variables with subjective vitality score. They are (**a**) The independent curvilinear association of green view index with subjective vitality score; (**b**) The independent curvilinear association of colorfulness index with subjective vitality score; (**c**) The independent curvilinear association of boundary enclosure with subjective vitality score; (**d**) The independent curvilinear association of aspect ratio with subjective vitality score. [Red lines represent point estimates of modeled score of revitalization, while the green background areas represent their 95% confidence intervals].

Meanwhile, the green view index should be designed in an appropriate threshold range. PET (Coef. = −0.239; p < 0.001) was negatively associated with the psychological restoration benefits experienced by the senior park visitors (Table 5). The relationship was more prominent in respondents with a PET of more than 16 °C (Figure 7e). In other words, planting trees with a high canopy cover is very important because it can significantly reduce PET, thus improving older adults’ thermal comfort and restoring them after mental fatigue during the autumn season in Tokyo.

### 3.3. Effects of Individual and Environmental Features on Subjective Vitality Benefits

The multilevel regression model was also used to explore the variables related to the subjective vitality benefits experienced by the older adults after visiting SPUGS (Table 6). Model 4 was a null model which showed that the environmental variables of the SPUGS could explain a significant portion of the variance in the respondents’ subjective vitality restoration (38.4%, ICC = 0.384). Model 5 showed similar results when compared with the ROS score. Respondents over 70 tended to report gaining higher subjective vitality benefits from the SPUGS (Coef. = 0.291; p < 0.01) (Coef. = 0.524; p < 0.001) as age increased. Female respondents reported higher levels of subjective vitality than male respondents (Coef. = 0.293; p < 0.05). The total time spent in the SPUGS was positively related to the reported levels of subjective vitality. When the older adults constantly stayed in one space for more than 30 min, the subjective vitality restoration scores continued to increase by 0.550 (Coef. = 0.550; p < 0.01). It was found that those respondents who visited the neighborhood parks for companionship would experience more vitality restoration benefits than those who visited to exercise (Coef. = 0.501; p < 0.001).

After adding the environmental features of the SPUGS, the individual features still correlated with the subjective vitality restoration benefits for older adults (Table 6). Among the environmental variables, colorfulness index (Coef. = 12.287; p < 0.001) had the strongest effect on the older adult’s vital recovery benefits. There is a linear fitting curve between colorfulness index and vitalizing effects (Figure 7b). Green view index (Coef. = −2.329; p < 0.001) was found to have a negative effect on the revitalization benefits of elderly respondents in SPUGS. Unlike the curve fitting of the ROS score, the curve between the green view index and the subjective vitality score of the older adults showed an approximately linear relationship (Figure 7a). Boundary enclosure (Coef. = 0.598; p < 0.001), and aspect ratio (Coef. = 0.485; p < 0.001) were positively related to our respondents’ subjective vitality score. However, the prediction curve showed a downward trend once the boundary enclosure reached 2 and the aspect ratio reached 1 (Figure 7c,d).

Unlike the regression results of the ROS score, the number of seats in the SPUGS could not impact the senior’s vitality benefits. By contrast, SPUGS without water feature (Coef. = −3.158; p < 0.001) positively affected the vitality restoration score. Furthermore, SPUGS with unpaved roads (Coef. = −2.806; p < 0.001) had a significant effect. It was also revealed that the PET did not correlate with the vitality benefits experienced by the senior visitors.

## 4. Discussion

This study investigated the mental restoration effects of SPUGS environmental features in Tokyo using a multilevel regression model. Our study’s hypothesis, that spatial form, natural, facility, and thermal features would influence psychological effects, is supported, because significant relationships were confirmed regarding the ROS and self-reported vitality recovery score. Control variables (age, time spent, reason for visit) were confirmed to have affected the mental health restoration benefits gained by the older adults.

As expected, our findings revealed that there are significant differences in the effects of environmental features on the ROS score and subjective vitality restoration experienced by the older adults: Subjective vitality is hypothesized to reflect organismic well-being and, thus, should covary with both the psychological and somatic factors that impact the energy available to an individual [50]. Thus, our findings confirmed that the environmental features of SPUGS also influence the physical health recovery benefits received by older adults to a certain degree.

According to the influence intensity, the ROS score was significantly affected by the sky view index (Coef. = −7.906; p < 0.05), boundary enclosure (Coef. = 1.901; p < 0.01), green view index (Coef. = −4.800; p < 0.05), number of seats (Coef. = 0.502; p < 0.001), and the PET (Coef. = −0.239; p < 0.001). These results suggest a lower level of sky visibility was associated with higher restorative effects among the senior respondents. This could be explained by the fact that the sky view factor measures the amount of shade from trees, which can reduce the heat stress, and increase the vision greenery of older adults while exposed to the SPUGS. These two aspects could affect mental recovery by promoting physical activity [60], releasing tension [61], and recovering the directed attention [20] for older adults.

Furthermore, the seat quantity should be increased in SPUGS, and the seats could be arranged at the space boundary to increase the enclosure degree. A flower bed with seating and a tree pool can be appropriately added in the center of the SPUGS to increase green visibility, but the crown of the tree pool should not be too high to avoid blocking the sky at the top interface. Meanwhile, the visibility of the external space should be enhanced through gaps between the vegetation in order to provide senior visitors with a rich spatial form so that they are in a suitable place to “see the outside” instead of “being seen,” which may increase their sense of security [62]. As such, it is assumed that a synergy of greenery, relatively cool conditions, and a sensation of safety in SPUGS can offer older adults the opportunity for enhanced relaxation, thoughts, and attention restoration.

Among the natural features, the colorfulness index showed the strongest effect on the subjective vitality outcomes of the older adults compared with the green view index. This indicated that colors may be a key factor influencing the resident’s spatial aesthetic perception. People who perceive more color can also perceive more species [63]; therefore, colors in outdoor spaces act as a trigger for raising the senior’s subjective vitality arousals. The regression results also revealed that unpaved paths in a SPUGS correlate with the respondents’ subjective vitality outcomes. In the parks in our research, the unpaved roads were made of gravel roads, which fit better aesthetically into the parks’ ecosystems on the one hand but also exhibited a level of heat stress that is close to the comfort range (18.1–23.0 °C) on the other [64]. Aspect ratio and boundary enclosure influenced the subjective vitality of the senior respondents positively. This suggests that the design of SPUGS should be enclosed at the edges, and a combination of facilities and shrubs could offer seniors with protection [19,65].

With regard to the thermal conditions, PET showed a significant relationship with the mental restoration, meaning that in autumn, a lower PET could diminish stress, lower fatigue, and facilitate a positive mood, reported in the older age group, which is consistent with extant results for younger respondents in summer season [40]. The curvilinear graph (Figure 6e) showed that ROS becomes maximum for PET equals 5 °C, where the thermal perception is “cold”, whereas ROS becomes minimum at around PET equals 16 °C. The rationality of this conclusion can be supported by Gatterer et al. [66] that relatively cold weather may enhance endurance exercise capacity and exercise more efficiently, thus mitigating health issues and promoting the mental restoration of the older adults [67,68]. However, in the full revitalization model, PET showed no effect on the vitalizing effect. For vitality represents energy that one can harness or regulate for purposive actions [51], which requires that basic physical and psychosocial needs are supported [69]. It is either unrelated or negatively related to affect in terms of anger, anxiety, or arousal [49]. This conclusion indicated that, compared with the thermal characteristic, the environmental variables which contribute to the promotion of visual greenery, color richness, and safety are more important for the promotion of vitality for older adults.

Unexpectedly, the SPUGS with water features was correlated with lower subjective vitality recovery scores, which contradicts previous findings [22,32]. Three possible factors might explain these differences. First, water features could occupy too much leisure space in the already limited space within SPUGS. Second, due to Tokyo’s subtropical climate, water features can breed mosquitoes, which may carry viruses to nearby residents (e.g., Kita City Central Park). Third, in the research unit in Kita City Park and Nishigahara Minnano Park, the water in the artificial pool was drained in autumn, thus, attracting children to play. Therefore, some older adults complained during the investigation about the high noise levels because of the children.

### Limitations and Implications

This study had three limitations. First, previous studies have emphasized that soundscape, air pollution, building density, and wind strength could influence the mental health of older adults by acting as an environmental stressor [70,71,72]. Due to equipment and workforce limitations, this study did not consider all built-up environmental features; thus, the assessment of wind strength could be a perspective for future research. Second, the study failed to obtain comprehensive background information during the investigation, given that the Japanese are often reticent about sharing personal details such as their marital status and income, which may lead to bias in the conclusion. Third, we were unable to assess the daily green exposure for each respondent. The mental restoration benefits would accumulate with increased mobility, which means that we could not ignore the potential impact of other types of SPUGS in the senior respondents’ daily routes, such as roadside or rooftop green spaces. In future research, wearable devices should be combined with the traditional survey method to explore the overall daily green exposure of older adults [73].

## 5. Conclusions

This study developed a multilevel regression model to investigate the environmental features that directly affect the ROS score and subjective vitality benefits for senior visitors in SPUGS in Kita-Ku, Tokyo. These findings not only support the socioecological framework developed by Lachowycz and Jones [34] but also supplement the literature from a systemic perspective by highlighting the influence of spatial form, nature, facility, and thermal features on the restorative effects and vitalizing effects of older adults in typical high-density Asian countries. Our study provides an evidence base for landscape designers regarding the psychological benefits seniors can gain from the spatial form, quantity of greenery, and the facility quality in SPUGS. These findings can provide landscape designers with strategies to develop healthy-aging SPUGS. The study’s main results can be summarized as follows:

- Participants over 70 were more likely to gain revitalization benefits from the SPUGS. When the older adults remained in the SPUGS for more than 30 min, the ROS and self-reported vitality restoration scores increased significantly. Companionship was found to exert an influence on the psychological benefits gained during visits to the parks, which supports previous studies;
- The sky view factor strongly affected the older adults’ restorative effects. The boundary enclosure and green view index have a significant impact, which suggests that shrubs and trees should be combined to provide older adults with a greater sense of enclosure and security, as well as offer a more appropriate level of greenness. The green view index should be about 0.4. Spaces with a proper number of seats are more inclined to provide psychological recovery benefits;
- The vitalizing effects were significantly affected by the colorfulness index, green vision rate, water features, road paving, boundary enclosure, and aspect ratio. Older adults can benefit from vitality recovery when visiting SPUGS through the perception of aesthetic and space edge protection. In addition, water features should be avoided in SPUGS;
- PET was confirmed to have negative, direct effects on the restorative benefits of older adults within the range of 5–20 °C. The relationship was more prominent in older respondents with a PET of more than 16 °C.

## Figures and Tables

**Table 1:** Basic information about the selected parks.

Basic Information	Park A (Hakusanbori)	Park B (Nishigahara Minnano)	Park C (Kita City Central)
Classification	Neighborhood park	City Block Park	Park
Area size (m^2^)	2500	21,900	79,200
Neighborhood aging rate (%)	36.27	25.10	21.00
Green cover rate (%)	52.50	59.70	72.80
Lawn-grass cover rate (%)	4.20	28.00	14.40

**Table 2:** Microclimate measurements and measurement instruments.

Microclimate Factors	Instruments	Height (m)	Range	Accuracy
Air temperature $\left(T_{a}\right)$	Temperature sensor IC (RTR503)	1.1	0–55 °C	±0.3 °C
Relative humidity (RH)	Humidity sensor IC (RTR503)	1.1	10–95%	±5% RH
Wind velocity (v)	Three-cup type wind speed meter (DT-187)	1.1	0.5–30 m/s	±0.2 m/s
Global temperature $\left(T_{g}\right)$	40 mm ping-pong ball with a class 1 type *T*-type thermocouple	1.1	−40–125 °C	±0.5 °C

**Table 3:** Study participants’ characteristics.

Variables	Full Sample (N = 202)	Sex	
		Male (N = 127)	Female (N = 75)
**Age**			
60–69	54 (26.73%)	31 (24.41%)	23 (30.67%)
70–79	104 (51.49%)	68 (53.54%)	36 (48.00%)
≥80	44 (21.78%)	28 (22.05%)	16 (21.33%)
**Health status**			
Very healthy	125 (61.88%)	80 (62.99%)	45 (60.00%)
Healthy	72 (35.64%)	43 (33.86%)	29 (38.67%)
Poor	5 (2.48%)	4 (3.15%)	1 (1.33%)
**Length of residency**			
<1 year	1 (0.50%)	1 (0.79%)	0
1–3 years	11 (5.45%)	8 (6.30%)	3 (4.00%)
>3 years	169 (83.66%)	103 (81.10%)	66 (88.00%)
**Visit frequency**			
1–2 times per week	121 (59.90%)	82 (64.57%)	39 (52.00%)
3–4 times per week	81 (40.10%)	45 (35.43%)	36 (48.00%)
**Distance from home**			
Less than 10 min	114 (56.44%)	69 (54.33%)	45 (60.00%)
10–20 min	56 (27.72%)	36 (28.35%)	20 (26.67%)
More than 20 min	32 (15.84%)	22 (17.32%)	10 (13.33%)
**Time spent**			
Less than 10 min	14 (6.93%)	8 (6.30%)	6 (8.00%)
10–30 min	54 (26.73%)	29 (22.83%)	25 (33.33%)
Over 30 min	134 (66.34%)	90 (70.87%)	44 (58.67%)
**Reason for visit**			
Exercise	85 (42.08%)	56 (44.09%)	29 (38.67%)
Socializing	23 (11.39%)	13 (10.24%)	10 (13.33%)
Relaxation	55 (27.23%)	37 (29.13%)	18 (24.00%)
Companionship	30 (14.85%)	14 (11.02%)	16 (21.33%)
Passing by/living near	9 (4.46%)	7 (5.51%)	2 (2.67%)
**Clothing insulation (SD)**	1.05 (0.18)	1.04 (0.18)	1.06 (0.18)
**PET (°C) (SD)**	15.24 (4.32)	14.97 (4.50)	15.75 (3.94)
**ROS score (SD)**	33.91 (4.00)	34.03 (4.18)	33.69 (3.70)
**Subjective vitality score (SD)**	5.13 (0.95)	5.06 (0.93)	5.25 (0.99)

**Table 4:** The environment feature variables in the research SPUGS.

	a1	a2	b1	b2	b3	c1	c2	c3	c4	c5	c6
**Skyview factor (mean)**	0.376	0.489	0.528	0.454	0.345	0.355	0.045	0.310	1.000	0.104	0.047
**Aspect ratio**	0.375	1.200	0.840	0.610	0.900	4.000	4.000	0.280	0.030	6.670	6.470
**Boundary enclosure**	1	2	1	2	3	3	4	3	4	2	2
**Green view index**	0.430	0.033	0.004	0.375	0.094	0.569	0.564	0.670	0.110	0.418	0.370
**Colorfulness index**	25	64	10	30	50	15	20	8	10	7	8
**Water facility**	No	Yes	No	No	Yes	No	No	No	No	No	Yes
**Number of seats**	4	2	1	4	2	2	0	2	6	2	4
**Road pavement**	No	Yes	Yes	No	Yes	Yes	Yes	No	No	Yes	No

**Table 5:** Multilevel regression results of ROS score.

Fixed Effects	Coefficient (SE)		
	Null Model 1	Model 2	Model 3
**Age (60–69 as reference)**			
70–79		0.199 (0.554)	0.320 (0.527)
Above 80		1.197 * (0.672)	1.018 (0.637)
**Sex (male as reference)**			
Female		−0.521 (0.472)	−0.669 (0.467)
**Health status (poor as reference)**			
Healthy		−0.912 (1.809)	−0.277 (1.690)
Not very healthy		−2.107 (1.826)	−1.715 (1.701)
**Length of residency (less than 1 year as reference)**			
1–3 years		1.549 (3.161)	1.658 (2.934)
More than 3 years		0.660 (3.012)	0.541 (2.778)
**Visit frequency (1–2 times per week as reference)**			
3–4 times per week		−0.048 (0.492)	0.107 (0.494)
**Time spent**			
10–30 min		2.716 ** (0.941)	2.971 ** (0.945)
More than 30 min		3.313 *** (0.887)	3.196 *** (0.893)
**Reason to visit (exercise as reference)**			
Socializing		0.862 (0.773)	0.531 (0.750)
Relaxation		0.714 (0.693)	0.636 (0.606)
Companionship		3.405 ***(0.725)	2.295 *** (0.722)
Passing by/living near		−0.292 (1.150)	0.313 (1.132)
**Access feature (less than 10 min walking as reference)**			
10–20 min walking			−0.018 (0.504)
More than 20 min walking			−0.380 (0.777)
**Spatial form features**			
Sky view factor			−7.906 * (3.346)
Boundary enclosure			1.901 ** (0.665)
Aspect ratio			−0.319 (0.386)
**Natural features**			
Green view index			−4.800 * (2.791)
Colorfulness index			−3.681 (6.326)
Water features (no as reference)			−2.226 (2.105)
**Facility features**			
Number of seats			0.502 *** (0.110)
Pavement (unpaved as reference)			−1.316 (1.788)
**Thermal feature**			
PET			−0.239 *** (0.066)
Intercept	34.236 *** (0.610)	31.233 *** (3.778)	33.940 *** (4.479)
Random effects			
ICC	0.183	0.249	9.13e-20
AIC	1106.4	953.9	872.959
Log likelihood	−550.200	−459.945	−408.480

* *p* < 0.05; ** *p* < 0.01; *** *p* < 0.001. SE: standard error; ICC: Interclass correlation efficient; AIC: Akaike information criterion.

**Table 6:** Multilevel regression results of subjective vitality benefits.

Fixed Effects	Coefficient (SE)		
	Null Model 4	Model 5	Model 6
**Age (60–69 as reference)**			
70–79		0.291 ** (0.118)	0.262 * (0.112)
Above 80		0.524 ***(0.142)	0.480 ***(0.135)
**Sex (male as reference)**			
Female		0.293 ** (0.100)	0.181 (0.099)
**Health status (poor as reference)**			
Healthy		0.569 (0.383)	0.531 (0.359)
Not very healthy		0.316 (0.386)	0.226 (0.361)
**Length of residency (less than 1 year as reference)**			
1–3 years		−0.375 (0.669)	−0.149 (0.623)
More than 3 years		−0.167 (0.638)	−0.090 (0.589)
**Visit frequency (1–2 times per week as reference)**			
3–4 times per week		−0.024 (0.105)	−0.010 (0.105)
**Time spent**			
10–30 min		0.207 (0.199)	0.292 (0.200)
More than 30 min		0.550 ** (0.188)	0.539 ** (0.189)
**Reason to visit (exercise as reference)**			
Socializing		−0.205 (0.166)	−0.154 (0.159)
Relaxation		0.137 (0.152)	0.177 (0.128)
Companionship		0.501 ***(0.154)	0.389 ** (0.153)
Passing by/living near		−0.311 (0.245)	−0.153 (0.240)
**Access feature (less than 10 min walking as reference)**			
10–20 min walking			−0.001 (0.107)
More than 20 min walking			−0.011 (0.165)
**Spatial form features**			
Sky view factor			−1.273 (0.710)
Boundary enclosure			0.598 *** (0.141)
Aspect ratio			0.485 *** (0.082)
**Natural features**			
Green view index			−2.329 *** (0.592)
Colorfulness index			12.287 *** (1.342)
Water features (no as reference)			−3.158 *** (0.500)
**Facility features**			
Number of seats			0.024 (0.023)
Pavement (unpaved as reference)			−2.806 *** (0.379)
**Thermal feature**			
PET			−0.005 (0.014)
Intercept	4.913 *** (0.190)	3.761 *** (0.812)	1.994 * (0.950)
Random effects			
ICC	0.384	0.453	2.63e-17
AIC	473.416	399.693	345.839
Log likelihood	−233.708	−182.847	−144.919

* *p* < 0.05; ** *p* < 0.01; *** *p* < 0.001. SE: standard error; ICC: Interclass correlation efficient; AIC: Akaike information criterion.

**Figure A1:**
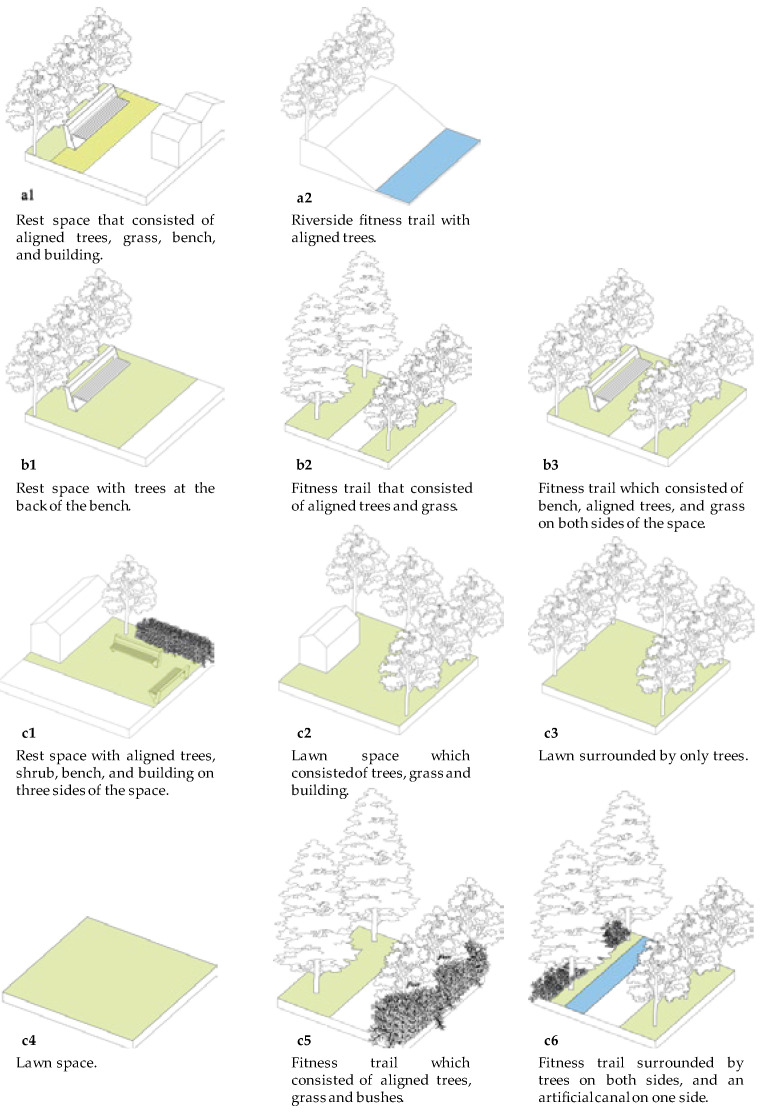
Typologies of the 11 SPUGS. The type code start with an “a” is the SPUGS divided from Hakusanbori Park; The type code start with a “b” is the SPUGS divided from Nishigahara Minano Park; The type code start with a “c” is the SPUGS divided from Kita City Park.

**Figure A2:**
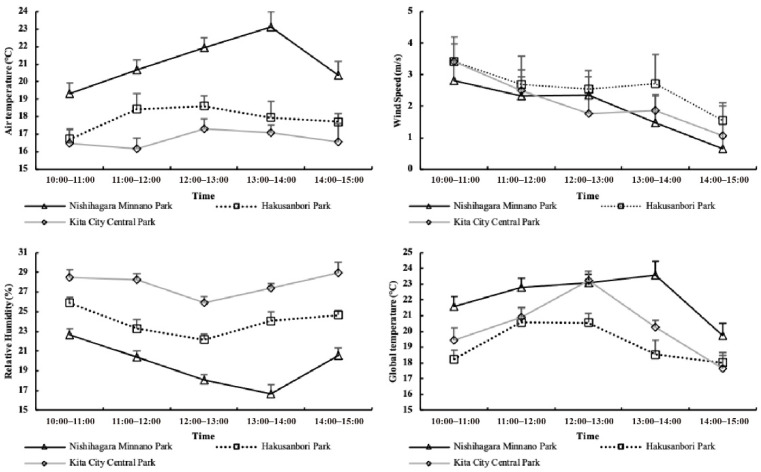
Microclimate variables measured in sunshine areas.

**Figure A3:**
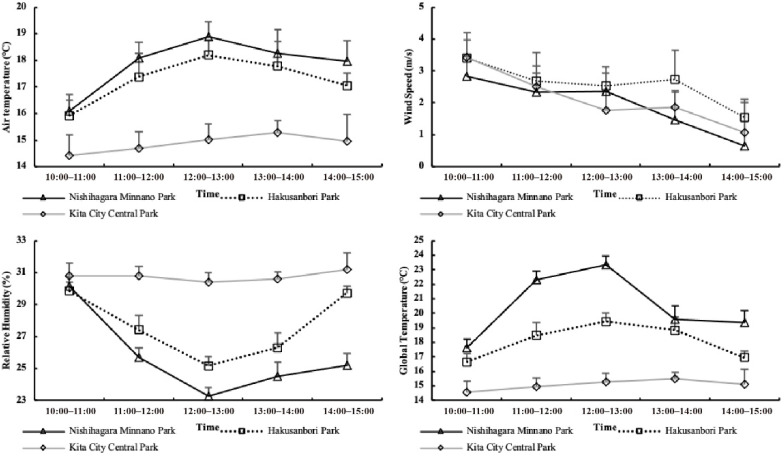
Microclimate variables measured in shade areas.

## Data Availability

Not applicable.

## Nomenclature

SPUGS	Small Public Urban Green Space
UGS	Urban Green Space
ART	Attention Restoration Theory
SRT	Stress Reduction Theory
PET	Physiological Equivalent Temperature
ROS	Restoration Outcome Scale
